# EEG-Based Detection of Mild Cognitive Impairment Using DWT-Based Features and Optimization Methods

**DOI:** 10.3390/diagnostics14151619

**Published:** 2024-07-26

**Authors:** Majid Aljalal, Saeed A. Aldosari, Khalil AlSharabi, Fahd A. Alturki

**Affiliations:** Department of Electrical Engineering, College of Engineering, King Saud University, Riyadh 11421, Saudi Arabia; dosari@ksu.edu.sa (S.A.A.); kabdulghani@ksu.edu.sa (K.A.); falturki@ksu.edu.sa (F.A.A.)

**Keywords:** EEG channel selection, DWT, machine learning, MCI, multi-objective optimization

## Abstract

In recent years, electroencephalography (EEG) has been investigated for identifying brain disorders. This technique involves placing multiple electrodes (channels) on the scalp to measure the brain’s activities. This study focuses on accurately detecting mild cognitive impairment (MCI) from the recorded EEG signals. To achieve this, this study first introduced discrete wavelet transform (DWT)-based approaches to generate reliable biomarkers for MCI. These approaches decompose each channel’s signal using DWT into a set of distinct frequency band signals, then extract features using a non-linear measure such as band power, energy, or entropy. Various machine learning approaches then classify the generated features. We investigated these methods on EEGs recorded using 19 channels from 29 MCI patients and 32 healthy subjects. In the second step, the study explored the possibility of decreasing the number of EEG channels while preserving, or even enhancing, classification accuracy. We employed multi-objective optimization techniques, such as the non-dominated sorting genetic algorithm (NSGA) and particle swarm optimization (PSO), to achieve this. The results show that the generated DWT-based features resulted in high full-channel classification accuracy scores. Furthermore, selecting fewer channels carefully leads to better accuracy scores. For instance, with a DWT-based approach, the full-channel accuracy achieved was 99.84%. With only four channels selected by NSGA-II, NSGA-III, or PSO, the accuracy increased to 99.97%. Furthermore, NSGA-II selects five channels, achieving an accuracy of 100%. The results show that the suggested DWT-based approaches are promising to detect MCI, and picking the most useful EEG channels makes the accuracy even higher. The use of a small number of electrodes paves the way for EEG-based diagnosis in clinical practice.

## 1. Introduction

Dementia is characterized by a progressive loss of mental functions, including speech, memory, and cognition, which makes daily functioning difficult [1,2]. Mild cognitive impairment (MCI) is the early stage of Alzheimer’s disease and other forms of dementia. The primary distinction between MCI and dementia lies in the fact that the cognitive decline associated with MCI does not significantly impact daily functioning, whereas dementia does [3,4]. Additionally, individuals with MCI do not typically exhibit the personality changes frequently observed in those with dementia. Nevertheless, individuals with MCI have a heightened susceptibility to the onset of Alzheimer’s disease or other forms of dementia. Approximately 15–25% of patients with MCI progress to Alzheimer’s disease annually [5]. Although the US Food and Drug Administration recently approved a new drug for Alzheimer’s disease [6], a diagnosis of Alzheimer’s disease during MCI can significantly slow its progression using non-drug treatments. However, accurately diagnosing MCI, or Alzheimer’s disease, can be challenging due to the extensive range of tests involved. These tests typically include psychological evaluations (such as the Mini-Mental State Examination [MMSE]); blood tests; analysis of spinal fluid; neurological examinations; and imaging techniques such as magnetic resonance imaging (MRI) [7], computed tomography (CT) [8], and positron emission tomography (PET) [7,9].

Electroencephalography (EEG) is a non-invasive technique that measures the electrical variations generated by the activities of thousands of neurons in the brain. By placing electrodes on the scalp, these electrical potentials can be measured [10]. The spatial resolution of the EEG, which refers to how well it can identify the location of brain activity, is affected by the number of electrodes employed and their placement on the scalp. EEG recording systems are less time-consuming; more economical; portable; and have outstanding temporal resolution when compared to other neuroimaging techniques like MRI, CT, and PET [10]. This means that they can capture changes in brain activity with very high resolution over time. Advances in EEG signal analysis, combined with machine learning approaches, have demonstrated EEG’s effectiveness in automatically identifying various neurological disorders. These include epilepsy [11,12,13,14], autism spectrum disorder [11,13], Alzheimer’s disease [15,16], schizophrenia [17], Parkinson’s disease [18,19], major depressive disorder [20], and even emotion recognition [21]. However, there has not been extensive exploration of EEG’s potential in detecting MCI. According to a review study [22] that focused on the use of EEG for the diagnosis and progression assessment of Alzheimer’s disease, a substantial body of literature (48 studies) primarily focuses on distinguishing Alzheimer’s disease from healthy controls. Using resting-state EEG to differentiate MCI from healthy controls has received relatively less attention.

In what follows, studies that devoted attention to the use of EEG-based methods for automatic MCI identification are discussed. To forecast Alzheimer’s disease in its initial phase, the study [23] explored granger causality and stochastic event synchrony to extract the required features. To classify the resulting features, discriminant analysis (DA) is used. The researchers utilized a dataset containing 22 individuals with MCI and 38 healthy control individuals. They employed 21 channels to assess their methodology and achieved an impressive classification accuracy of 83%. In [24], a method to distinguish patients with MCI from those who are healthy controls by analyzing fundamental spectral features is introduced. The researchers employed a neuro-fuzzy algorithm in conjunction with a K-nearest neighbor (KNN) classifier to classify the resulting features from a group of 11 patients with MCI, 16 healthy control individuals, and 19 EEG channels. Each channel’s signal was divided into 1 s segments, with each segment overlapping by 50% to reach an accuracy score of 88.89%. In [25], the authors of [24] expanded the participants to include 29 individuals with MCI and 32 healthy controls. They introduced a method for analyzing EEG signals termed correlation-based label-consistent K-SVD (CLC-KSVD), which utilizes supervised dictionary learning. By applying brain-region-based classification, an accuracy score of 80% was achieved when the channels belonged to the left-temporal area. By using their suggested approach to the dataset in [24], an 89% accuracy score was achieved with the identical cerebral area. In [26], the KNN classifier was employed to classify power spectral features, achieving a classification accuracy score of 81.5% on the identical dataset examined in [24]. A set of features called relative power-based features (KERP) are introduced in [27] to classify individuals with MCI and healthy controls. The investigation involved recording EEGs from 24 patients with MCI and 27 healthy control subjects using 30 channels. The recorded EEGs were separated into segments of 3 s each, with a 50% overlap between consecutive segments. Prior to using the classifier, the researchers used Fisher’s method to select the retrieved features. These features that were chosen were then fed to the support vector machine (SVM) classifier, attaining 90.20% classification accuracy. Study [28] used the same dataset as [24], but eliminated five instances of healthy controls to equalize the ratio between patients with MCI and healthy control cases. For the purpose of enhancing the signal-to-noise ratio, the researchers in [28] utilized stationary wavelet transformation (SWT). Subsequently, they derived nine statistical features. After splitting the resulting feature vectors into three sets: 60% for training, 20% for validation, and 20% for testing, their employed SVM classifier attained 96.94% accuracy. In [29], auto-regressive and permutation entropy were utilized to extract features from the dataset given in [24]. These features were subsequently classified using a classifier called an extreme learning machine (ELM). Using this classifier and applying a 10-fold cross-validation strategy, an accuracy of 98.78% was obtained. In another study [30], long-short-term memory (LSTM)-based approaches were developed. The researchers in [30] produced 20 different LSTM models and assessed them on the dataset in [24]. They determined the most optimal model, which achieved an accuracy of 96.41% using five-fold cross-validation. In [31], various measures were investigated and combined to extract features for detecting MCI. A total of 1500 features were retrieved from the 32 channels. The dataset involved 21 individuals with MCI and 21 normal participants. In this study, the accuracy scores were assessed for symmetric combinations of two, four, six, and eight electrodes using an SVM classifier, trying to decrease the number of channels or electrodes used. The two-electrode configuration achieved a classification accuracy of 74.04%, while the four-electrode configuration achieved 82.43%. The six-electrode configuration achieved an accuracy of 86.28%, and the eight-electrode configuration achieved the highest accuracy of 86.85%. A study in [32] extracted a total of 425 features to represent the EEGs of 18 people with MCI and 16 healthy control subjects. These features included spectral, functional connectivity, and nonlinear aspects and were obtained from recordings made using 19 channels. The linear SVM model attained an accuracy of 99.4% using all feature sets and employing 10-fold cross-validation. A recent study [33] used the same dataset from [32], employing the discrete wavelet transform (DWT) leader to generate features. By applying the AdaBoostM1 method as a classifier, a 93.50% accuracy score was obtained. A more recent study [34] used the same dataset as in [25]. Empirical mode decomposition (EMD) decomposes EEG signals to generate features before computing them. With a KNN-based classification, an accuracy score of 97.60% was obtained.

Furthermore, several researchers have investigated methods for distinguishing between Alzheimer’s disease, MCI, and healthy controls. For instance, [35] used a dataset comprising 44 individuals, including 15 with dementia, 16 with MCI, and 13 healthy controls. The SVM-based classification was based on four conditions, including eye-open and eye-close resting states. The aim was to investigate the differentiation between MCI, dementia, and healthy controls. Eight measures were examined to extract features from 21 channels. A diagnostic accuracy of 84.1% was attained when differentiating between individuals with MCI and healthy controls using a 10-fold cross-validation strategy and analyzing resting state data with eyes open. For the 109 participants employed in [36], a decision tree (DT) classifier was used to assess Fourier and wavelet transforms. The participants had 19 channel EEG signals and were divided into three groups: 49 with Alzheimer’s disease, 37 with MCI, and 23 healthy controls. In the task of classifying MCI vs. healthy controls, the DWT achieved the best performance. This method resulted in an accuracy score of 83.3% when using holdout validation and 93.3% when using 10-fold cross-validation. In a recent study [37], 16 people with MCI, 8 people with Alzheimer’s disease, and 11 healthy controls were used to create features using DWT, PSD, and interhemispheric coherence. To achieve an accuracy of 95.50%, a bagged tree classifier was employed together with five-fold cross-validation. Another recent study [38] calculated the power intensity of EEG signals from 105 individuals, including 48 with Alzheimer’s disease, 37 with MCI, and 20 healthy controls, for both high- and low-frequency bands. Multiple classification tasks were examined using SVM, DT, and KNN classifiers. The KNN algorithm achieved a classification accuracy of 95% in the task of differentiating individuals with MCI from healthy controls, with a 10-fold cross-validation strategy.

Meticulously designing feature extraction can significantly enhance diagnosis accuracy. Furthermore, selecting the most relevant channels can improve the accuracy of the diagnosis. Several feature extraction methods have been proposed in the literature. However, there is still a need to introduce new and efficient methods for better representation of EEG signals. In addition, most studies in the literature utilize all of the EEG channels available in the datasets. Using all channels may not necessarily lead to improved efficiency or classification accuracy due to the presence of duplicate, irrelevant, or poor information in some channels [39]. In addition, reducing the number of EEG channels results in lower computational costs, a shorter preparation duration, and enhanced classification accuracy by reducing the likelihood of over-fitting that may occur when using all channels. Except for [31], the current efforts disregard the exploration of the most optimal combination of EEG channels. However, [31] only focuses on the use of two, four, six, and eight channels. Furthermore, the study is limited by the requirement for symmetric channel pairs, not considering the entire search space. This symmetric strategy disregards numerous two-channel subsets that have the potential to result in greater accuracy. This principle is also applicable to combinations including four, six, and eight channels. The authors of [31] employed the method of using symmetric channel pairings because of the inherent challenge of evaluating all possible combinations of two, four, six, or eight channels. In our recent study [40], we looked at how to use a non-dominated sorting genetic algorithm (NSGA-II) to reduce the number of EEG channels, along with feature extraction methods based on variational mode decomposition (VMD). The findings presented in [40] indicated the possibility of decreasing the number of channels without compromising classification accuracy. The present study is to confirm and validate the results in [40]. We achieve this by examining alternative approaches for EEG channel selection and more efficient methods for feature extraction. Therefore, the following is a summary of the present study’s contributions:

- Introducing efficient DWT-based feature extraction methods to generate reliable biomarkers for MCI detection. In these methods, only one of the adopted non-linear measures follows the DWT decomposition and reconstruction processes.
- Enhancing the detection accuracy by utilizing multi-objective optimization techniques to identify the most effective channel subsets. In addition to NSGA-II examined in [40], the present study explores NSGA-III as well as a particle swarm optimization (PSO)-based approach. We designed these three multi-objective optimization methods to solve a two-objective function problem: optimizing MCI detection accuracy and, simultaneously, decreasing the number of EEG channels.
- Additionally, greedy algorithms, including back-elimination (BE) and forward-addition (FA), are also explored for EEG channel selection.
- Investigating different machine learning algorithms and optimizing their parameters using multi-objective optimization methods.
- Evaluating the developed approaches using the public dataset from [24,26,28,29,30] and combining it with the dataset from [32,33] to ensure a balanced and larger subject count, akin to [25,34,40]. Furthermore, the results are validated with the most common validation type, k-fold cross-validation.

The paper is prepared as follows: Section 2 presents a description of the EEG datasets used, including the applied methods for preprocessing, feature extraction, and classification, as well as the proposed methods for EEG channel selection. Section 3 outlines the findings and analysis, while Section 4 summarizes the study and offers suggestions.

## 2. Materials and Methods

**Figure 1:**
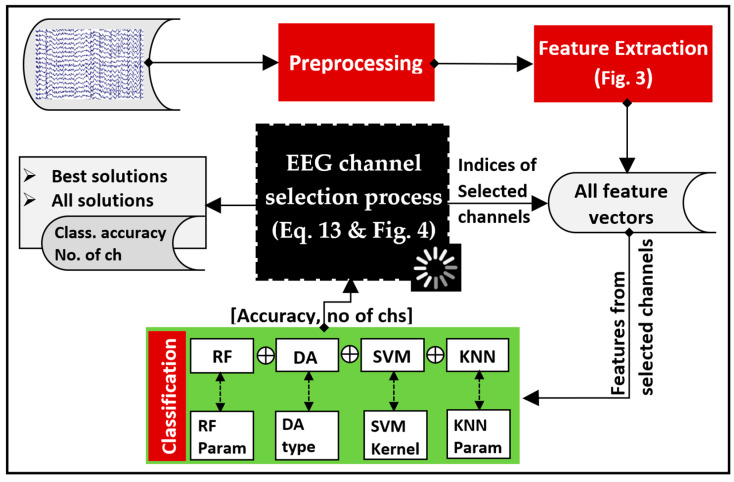
A high-level overview of procedures followed in the present study.

Figure 1 illustrates the stages of the EEG signal processing and the channel selection process using multi-objective optimization techniques. We investigated various approaches, including greedy algorithms and multi-objective optimization-based techniques, for EEG channel selection. The operations in Figure 1 can be briefly described as follows. Preprocessing removed artifacts from the raw EEG dataset. Preprocessed EEG signals were separated into equal-length, non-overlapping segments. Each segment was subsequently decomposed into various frequency bands using the DWT. Features were obtained from each sub-band using a non-linear measure. We investigated the use of several feature extraction measures, including different entropy measures as well as energy measures. The extracted feature values were collected into a feature vector for each channel in a segment. After obtaining all feature vectors, the classification was implemented in two scenarios: full-channel-based and selected channel-based classification. In the case of full-channel-based classification, the classification was implemented using all of the resulting feature vectors. Different classification algorithms, including KNN, linear discriminant analysis (LDA), discriminant analysis quadratic (QDA), SVM, and random forest (RF), were employed to compare their accuracies in identifying MCI feature vectors from those that are normal. In the case of selected channel-based classification, only the feature vectors belonging to the channels that had been selected were fed as input for the classifier. For channel selection, we compared several methods, including BE, FA, NSGA-II, NSGA-III, and PSO. In the case of multi-objective optimization methods (NSGA-II, NSGA-III, and PSO), a dynamic approach was employed to optimize two objectives: the classification accuracy and the number of channels needed for MCI classification. The subsequent sections provide details of each stage in the block diagram. 

### 2.1. Datasets and Pre-Processing

**Figure 2:**
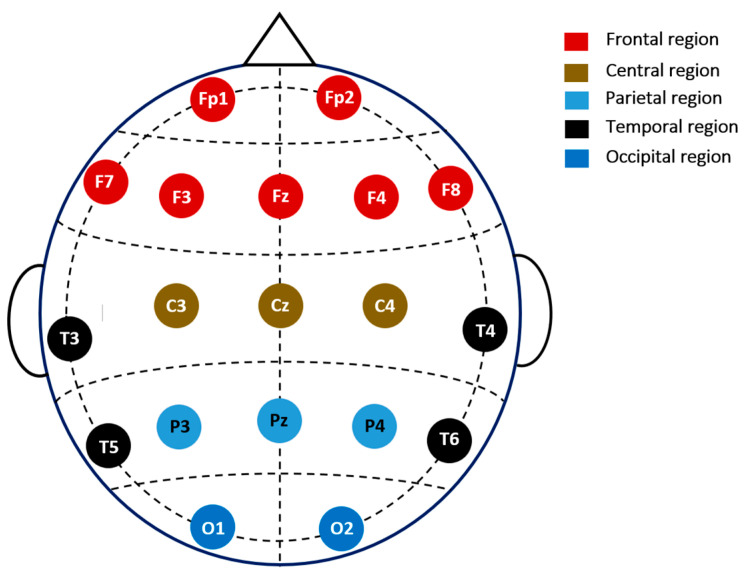
The 19 channels investigated in the current study and their corresponding locations.

The efficacy of the proposed approaches was evaluated by conducting experiments on two publicly accessible datasets [41]. The first dataset included 11 individuals with MCI and 16 healthy people, while the second dataset included 18 MCI patients and 16 healthy subjects. Following the methodology of [25,34,40], we combined these two datasets to create a more comprehensive and balanced dataset. This consolidated dataset consisted of 61 participants, all aged 55 or above, including 32 healthy individuals and 29 MCI patients. EEG recordings, participant recruitment, cognitive assessments, and other procedures were conducted in Isfahan, Iran, at Noor Hospital [42]. Individuals with a history of dementia, severe physical illnesses, substance misuse, head injuries, or serious mental illnesses were excluded. MCI was identified based on Mini-Mental State Examination (MMSE) scores within the 21–26 range, and scores above 27 were deemed normal. The Neuropsychiatry Unit Cognitive Assessment Tool (NUCOG) was also used to support the diagnosis of MCI, with a score range of 75–86.5 [43]. As described in [42], all subjects underwent EEG recordings in a quiet environment, lying down with their eyes closed in the morning. Nineteen EEG electrodes were placed according to the 10–20 International System, as depicted in Figure 2. A 32 channel digital EEG instrument (Galileo NT, EBneuro, Italy) was utilized to record the EEG data at a 256 Hz sampling rate, maintaining the electrode-to-skin resistance under 5 k Ohms. More details of the EEG recording can be found in [24,42]. EEG data were recorded for 30 min for each participant, but only the initial 10 min were used to avoid participant fatigue.

The EEG recordings underwent preprocessing, where a fifth-order band-pass Butterworth filter with a frequency range of 0.5–32 Hz was used. This filter effectively eliminated baseline artifacts and AC power-line noise. Visual inspection eliminated the remaining artifacts to guarantee a clean dataset. Subsequently, the signals were partitioned into equal segments, each with a size of ch×N, where ch is the number of channels and N represents the number of samples. In this study, each segment was 10 s in length. As previously mentioned, the study explored various methods with the aim of decreasing ch while simultaneously improving, or at the very least maintaining, the classification accuracy of MCI versus healthy controls (HC).

### 2.2. DWT-Based Features

#### 2.2.1. Decomposition and Reconstructing

**Figure 3:**
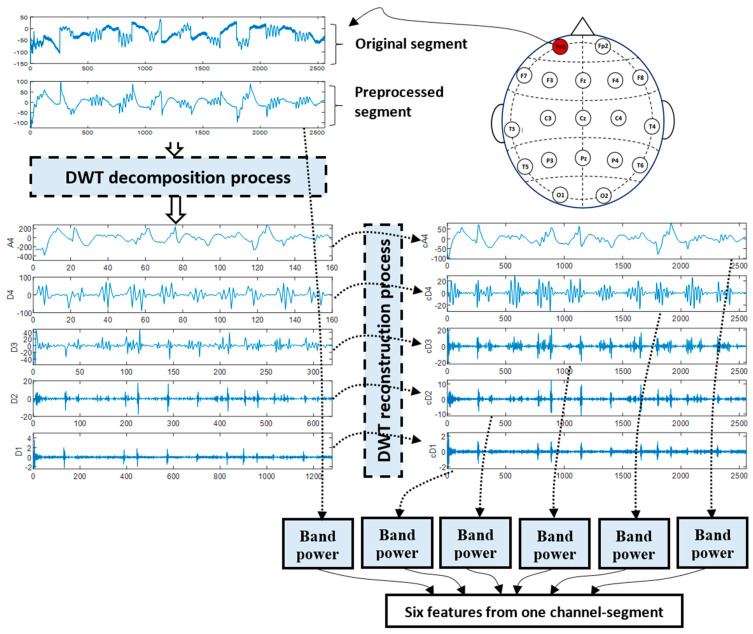
An illustrative example of DWT-based feature extraction from a 10 s segment (DWT+LBP).

In line with the methodology employed in [19], we utilized the DWT to decompose and reconstruct each channel’s segment, as illustrated in Figure 3. In the present study, the db4 mother wavelet was selected for the decomposition process. According to the review study in [44], this wavelet is widely used in EEG signal analysis. Initially, the preprocessed segmented signals from each channel were decomposed by DWT into four detail coefficients (D4, D3, D2, and D1) and one approximate coefficient (A4), corresponding to the theta, alpha, beta, gamma, and delta EEG sub-bands [10]. Subsequently, DWT was then used to reconstruct each wavelet packet (WP) signal separately from its corresponding coefficient, as shown in Figure 3. In other words, cA4 was reconstructed from A4, cD4 was reconstructed from D4, and so on. This reconstruction process was intended to improve the time resolution, especially at low frequencies. The reconstructed WP signals (cA4, cD4, cD3, cD2, and cD1) were anticipated to yield effective biomarkers for MCI identification, given their ability to capture both time and frequency information with high resolution.

#### 2.2.2. Computing Features

The subsequent step was to compute features from the reconstructed WP signals and the original segment, as illustrated in Figure 3. We compared the effectiveness of various feature types: band power, energy, and entropy. If $S\left(n\right)$ is a discrete signal (cA4, for example), n=1, 2, …, N, with N sample, the energy and band power of this signal are [45]

Energy (Eng):

(1) $$\mathrm{Eng}=\sum_{n=1}^{N}{\left|S\left(n\right)\right|}^{2}$$

Band power (BP):

(2) $$\mathrm{BP}=\log\left[\frac{1}{N}\sum_{n=1}^{N}{\left|S\left(n\right)\right|}^{2}\right]$$

For a given time series, a typical measure for assessing its complexity, regularity, and statistical quantification is entropy. It has been shown to be an effective biomarker for a wide range of brain disorders, such as epilepsy [46], attention deficit hyperactivity disorder [47], and autism [48]. This inspires us to consider this measure as a feature extractor to detect MCI. Instead of extracting the feature from the preprocessed segments, as in the just-mentioned studies, we suggest extracting the feature from WP signals, which may help create suitable biomarkers for MCI detection.

We examined several entropy measures, which are defined next. If k represents the number of unique values in the discrete signal $S\left(n\right)$ and $\;x_{j}$ represents the probability frequency of the *i*th unique value, then entropy measures are given by [49]

Threshold entropy (ThEn)

(3) $$\mathrm{ThEn}=\#\left\{i\;\mathrm{such}\;\mathrm{that}\;\left|x_{i}\right|>\alpha \right\}$$

where ThEn represents the number of time instants for which the signal exceeds a predetermined threshold α. According to [49], the threshold α should be lower than 1. In this study, it was empirically set to 0.2.

Norm entropy (NoEn)

(4) $$\mathrm{NoEn}=\sum_{i=1}^{k}{\left|x_{i}\right|}^{p}\;$$

where p is the power of the entropy and must be such that 1 ≤  P. In this study, it was selected to be 1.1.

Sure entropy (SuEn)

(5) $$\mathrm{SuEn}=k-\#\left\{i\;\mathrm{such}\;\mathrm{that}\;\left|x_{i}\right|\leq £\right\}+\sum_{i}\min\left(x_{i}^{2},{\;£}^{2}\right)$$

where $\#\left\{i\;\mathrm{such}\;\mathrm{that}\;\left|x_{i}\right|\leq £\right\}$ represents the number of time instants for which the signal exceeds a predetermined threshold £, and usually £>2. In the present study, it was selected to be 3.

Log energy entropy (LogEn)

(6) $$\;\mathrm{LogEn}=\sum_{i=1}^{k}log{\left|x_{i}\right|}^{2}\;\;\;\;\;\;$$

Transformation-Shannon entropy (T-ShEn)

(7) $$\mathrm{TShEn}=\frac{1}{k}\sum_{i=1}^{k}{\left|x_{i}\right|}^{2}log{\left|x_{i}\right|}^{2}\;\;\;\;\;\;\;$$

Accordingly, one of the adopted measures (Equations (1)–(7)) was employed to extract six features: five from WP signals and the sixth from the original signal, as illustrated in Figure 3. Accordingly, the total number of features acquired from one segment was equal to 6×ch, resulting in a single feature vector. This process was repeated over all data segments to finally obtain a collection of feature vectors (feature matrix) representing MCI and HC cases. The next step was to implement the classification (either a full-channel-based classification or a selected-channel-based classification).

### 2.3. Classification

In this work, we considered several classification techniques: KNN, SVM, LDA, QDA, and bagging-based RF. The aim was to find classifiers that provide superior MCI versus HC classification results. Details of these techniques can be found in [50,51,52,53] and the references therein. Most of these techniques have hyperparameters that affect the way the classifier performs. In the case of full-channel-based classification, arbitrary settings were applied. On the other hand, in the case of selected-channel-based classification, the classifiers’ parameters were included in the optimization process to enhance the classification accuracy. In this case, the KNN number of neighbors (K) was optimized within a range of 1–10 neighbors. The SVM was evaluated using three distinct kernels: linear, polynomial, and radial basis function (RBF), while the RF was evaluated by varying the tree depths from 1 to 35.

### 2.4. Performance Evaluation

In order to evaluate the effectiveness of the developed model (feature extraction + classification), a 10-fold cross-validation strategy was employed. This strategy involves randomly separating the complete data into ten subgroups, with one subgroup dedicated to validation (testing) and the remaining nine subgroups used for training [54]. The test and training subgroups underwent them ten times (10-fold). Each time, a single classification measure was generated by computing the classification accuracy (CA), sensitivity, specificity, and F-score using Equations (8)–(12) and then averaging the results over the ten rounds.

(8) $$CA=\frac{N_{correct}}{N_{total}}\times 100\%\;$$

(9) $$Sensitivity=\frac{TP}{TP+FN}\times 100\%\;\;\;\;$$

(10) $$Specificity=\frac{TN}{TN+FP}\times 100\%\;\;$$

(11) $$F-score=2\times \frac{precision\times recall}{precision+recall}\times 100\%$$

where *N*_*correct*_ is the number of feature vectors that are correctly classified, *N*_*total*_ is the total number of feature vectors that need to be classified, *TP* is true positives, *TN* is true negatives, *FN* is false negatives, and *FP* is false positives. The capability of a classification model to correctly recognize individuals with the disease is indicated by the *sensitivity*, also known as recall or true positive rate (TPR). On the other hand, the capability of a classification model to accurately recognize people without the disease is indicated by the *specificity*, also known as the true negative rate (TNR) [55]. The *precision* is given by

(12) $$Precision=\frac{TP}{TP+FP}\times 100\;\;$$

### 2.5. EEG Channel Selection

Not all 19 EEG channels are equally important, since some may contain redundant or useless information [39]. Therefore, channel selection can help reduce the computing burden of signal processing, expedite the preparation time, enhance classification accuracy, and identify brain regions that produce task-dependent activity. In this study, we investigated the efficacy of several approaches for channel selection. These approaches are explained next. 

#### 2.5.1. Greedy Algorithms

These algorithms are simple and quick methods for assessing the most important parameters or features to obtain the best results [56]. In these methods, optimization decisions (e.g., channel inclusion or exclusion) are made at each iteration based on assessing a single parameter’s effect, leading to solutions that are generally suboptimal. Two greedy EEG channel selection algorithms are presented next.

##### Back-Elimination Algorithm

The back-elimination, or BE, algorithm is commonly used for feature subset selection [57,58]. In this study, BE was employed at each iteration to select the best channel combination from the available 19 channels. To achieve this goal, BE requires 19 iterations. In the first iteration, BE randomly eliminates one channel and then calculates the classification accuracy using the remaining 18 channels. The eliminated channel is returned back to the channel pool, and the operation is repeated. Since there are 19 times for a channel that can be excluded, one accuracy value is obtained each time, and thus a set of accuracy values is generated in the first iteration: $CA_{1,1},\;CA_{1,2},\ldots ,\;CA_{1,19}$. This iteration ends by selecting the 18 channel subset ch_selected1 with the highest accuracy $MaxCA_{1}=Max\;\left(CA_{1,1},\;\;CA_{1,2},\ldots ,\;CA_{1,19}\right)$. In the second iteration, the same process is applied to the 18 channel subset $ch\_selected_{1}$ found in the first iteration. This will result in a 17 channel subset $ch\_selected_{2}\;$ with the maximum accuracy value $\;MaxCA_{2}=Max(CA_{2,1},\;CA_{2,2},\ldots ,\;CA_{2,18}$). Since every iteration removes one channel, only a single channel will remain after finishing all iterations. The final outputs of the BE are two vectors; the maximum accuracy scores, $[MaxCA_{1},\;MaxCA_{1},\ldots ,\;MaxCA_{19}],$ are included in the first vector, while the second vector contains the corresponding channel subsets, [$ch\_selected_{1},\;ch\_selected_{2},\;\;\ldots ,\;\;{{ch}_{selected}}_{19}$]. For the available 19 channels, the classification accuracy is evaluated 19 × 20/2 = 190 times.

##### Forward-Addition Algorithm

The forward-addition, or FA, algorithm operates similarly to BE but in reverse order by progressively adding channels instead of removing them. For the 19 channels, 19 iterations are needed to finish the EEG channel selection process, like BE. In the first iteration, the classifier only considers features available from one channel, while the remaining are omitted. Since there are 19 possible channels to choose from, one accuracy score is obtained for each of them; thus, a set of accuracy values is generated in the first iteration: $CA_{1,1},\;CA_{1,2},\ldots ,\;CA_{1,19}$. The highest accuracy, $MaxCA_{1}=Max\;\left(CA_{1,1},\;CA_{1,2},\ldots ,\;CA_{1,19}\right)$, is preserved along with its corresponding channel, $ch\_selected_{1}$. In the second iteration, the same process is repeated, in which one of the 18 remaining channels is frequently added to $ch\_selected_{1}$ to form 18 two-channel subsets. For each two-channel subset, one accuracy score is obtained. The subset $ch\_selected_{2}$, with the highest accuracy $MaxCA_{2}=Max\;\left(CA_{2,1},\;CA_{2,2},\ldots ,\;CA_{2,18}\right)$ is preserved (second local optimal). This finishes the second iteration. Since one channel is added at each iteration, the size of the last subset becomes 19 in the 19^th^ iteration. Similar to BE, FA finishes its process with two vectors; the maximum accuracy scores, $[MaxCA_{1},\;MaxCA_{1},\ldots ,\;MaxCA_{19}],$ are included in the first vector, while the second vector contains the corresponding subset of channels, [$ch\_selected_{1},\;\;ch\_selected_{2},\;\ldots ,\;ch\_selected_{19}$]. Like BE, the classification accuracy is computed 190 times.

#### 2.5.2. Multi-Objective Optimization Algorithms

A multi-objective optimization problem (MOOP) necessitates the concurrent optimization of multiple objective functions. The required optimization may be maximizing the objective functions or minimizing them. With MOOP, optimizing one of the objectives can come at the expense of another, especially when their functions are conflicting. In such problems, a solution is considered feasible if it complies with all the constraints (in the case of constrained MOOP) and optimal if it yields the best result [59]. In this work, we considered the optimization of a two-objective problem using two multi-objective optimization algorithms: one based on the (NSGA) and the other based on PSO. These two algorithms and the problem to be optimized are briefly presented next.

##### NSGA-II and NSGA-III

The genetic algorithm (GA) is a search heuristic inspired by Charles Darwin’s theory of natural evolution. It is commonly employed to tackle complex optimization and search problems. In GAs, a population of potential solutions, referred to as chromosomes, is maintained, with each chromosome comprising a set of parameters known as genes [60]. GAs typically include population initialization, fitness function computation, crossover, mutation, survivor selection, and termination criteria to return the best solutions [61].

In a MOOP, a solution that is Pareto-optimal, or non-dominated, surpasses all other solutions (dominated solutions). The initial version of NSGA utilizes a non-dominated sorting selection approach to pinpoint potential candidates. It also maintains steady sub-populations of superior points, termed the Pareto front, via a method known as the niche method [62]. However, this version faced challenges that were addressed in the second version (NSGA-II). These challenges included concerns regarding computational complexity, population diversity, and its non-elitist approach. In NSGA-II [63], the computational cost was significantly reduced from $O\left(PQ^{3}\right)$ to $O\left(PQ^{2}\right),$ where P represents the population size and Q represents the number of objectives. Furthermore, the elitism approach was applied, which involves comparing the present population with the best non-dominated solutions that were previously identified [63]. Notably, unlike standard genetic algorithm parameters such as population size, termination parameter, crossover, and mutation probabilities, the NSGAII elitism approach does not require the configuration of any new parameters.

NSGA-III, the third version of NSGA [64], is effective at solving optimization problems with two to fifteen objectives. It adheres to the NSGAII structure but makes use of a collection of predetermined reference points that emphasize population members that are not dominant but are similar to the provided set [64]. A specified set of reference points is employed to ensure variation in the produced solutions. The reference points for NSGA-III are typically located on a normalized hyperplane that intersects all objective axes and is equally inclined to each [65].

##### Multi-Objective Particle Swarm Optimization (MOPSO)

The movement of a flock of birds as they search for food served as the basis for the PSO algorithm [66,67]. Each particle in the d-dimensional search space moves in accordance with its interactions with other particles to determine the optimum food’s position. In each iteration, each particle moves closer to the food until the most optimal, or final, solution is found. With each iteration, each particle’s current position and velocity in the swarm (population) are updated to serve as identification. Similar to GA, PSO begins by randomly initializing the population. Unlike GA operators (crossover and mutation), PSO solutions are given randomized velocities to explore the search space. Each feasible solution is referred to as a “particle” in PSO, whereas it is a “chromosome” in GA. MOPSO is an extension of the PSO algorithm designed to address multi-objective optimization problems [68]. MOPSO aims to find a set of solutions that represent the trade-off between different objectives, known as the Pareto front. Similar to PSO, particles in MOPSO share information and move towards both the global best particles and their own personal (local) best memory. However, unlike PSO, MOPSO involves multiple criteria to determine and define the best solutions, both globally and locally [68]. 

##### The Optimization Problem and Variables

The task at hand is to determine the most relevant and suitable channels, with a focus on either boosting or preserving the accuracy of MCI classification. This task is achieved by solving the following problem:

The problem to be solved is to identify the most relevant and appropriate channels while increasing or maintaining MCI classification accuracy. Equation (13) represents the problem with two objective functions.

(13) $$\begin{array}{ll} & \mathrm{Maximize}\;\;\;\;\;\;\;\;\;\;\;\;\;\;CA\left(channels,Param\right)\\ & \mathrm{Minimize}\;\;\;\;\;\;\;\;\;\;\;\;\;\;no\_ch\\ & \mathrm{Subject}\;\mathrm{to}\;\;\;\;\;\;\;\;\;\;\;\;\;CA\leq 100\\ & \;\;\;\;\;\;\;\;\;\;\;\;\;\;\;\;\;\;\;\;\;\;\;\;\;\;\;no\_ch\geq 1 \end{array}$$

As presented in Equation (13), the problem includes two objective functions: CA, which represents the average classification accuracy of MCI against HC computed based on Equation (8), and no_ch, which represents the number of channels. Param is a variable used to optimize the classifier’s parameters. 

**Figure 4:**
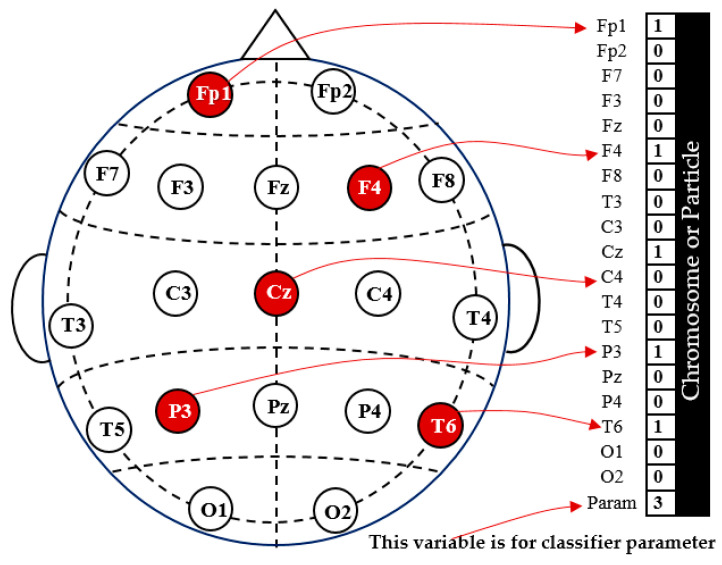
An illustration of NSGA (or PSO) channel representation in a chromosome (or particle).

It is required to organize the dataset and define the variable representation to solve the problem identified in Equation (13) using NSGA or PSO. As the average classification accuracy, CA, is a function of the channels, $ch_{1}$, $ch_{2}$,…, $ch_{19}$ and Param, they can be represented by one chromosome with 20 genes or one particle with 20 dimensions (one solution with 20 variables). In this particular representation, the first 19 variables are dedicated to represent the 19 channels (as illustrated in Figure 4), and the final one, param, is for the classifier parameter. Each of the first 19 variables has a binary value, which can be either “1” or “0”. A value of “1” indicates that the channel is selected, while a value of “0” means that the channel is excluded from the classification process. Figure 4 shows an example of the binary representation of the investigated channels. The last variable is dedicated to optimizing a parameter of the classifier used. For the KNN classifier, to represent the neighbor’s numbers, K, the parameter Param may have values from 1 to 10. With SVM, the Param variable is used to choose the kernel type: linear is represented by 1, polynomial is represented by 2, or RBF is represented by 3. Similarly, for the DA classifier, the Param variable represents the type of discriminant analysis: linear is represented by 1 or quadratic is represented by 2. In the RF classifier, the Param variable is responsible for optimizing different tree depths, which can range from 1 to 35.

**Figure 5:**
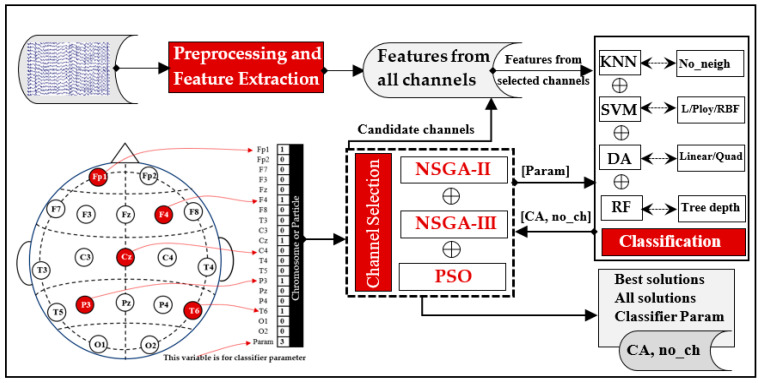
EEG channel selection process based on NSGA or PSO for MCI classification.

Finally, the complete process is presented in Figure 5, and its explanation is summarized as follows:

1. Initially, all channels’ signals are subjected to the preprocessing stage. Following this, the signals are decomposed and subsequently reconstructed using DWT (refer to Figure 3).
2. Features are then calculated using a non-linear measure. In this study, we compared several non-linear measures given by Equations (1)–(7).
3. From this point on, NSGA-based or PSO-based algorithms handle the main process of optimizing the selected channels as well as the classifier’s parameters. The process begins with creating an initial population representing possible solutions (chromosomes or particles). The values in each possible solution represent a set of selected EEG channels (which channels are included or excluded) and the value of the classifier’s parameter.
4. The features that belong to the selected channels (channels with a value of 1 in the generated solution) are fed as inputs for the adopted classifier, while the other features are not.
5. For each solution in the population (each chromosome in NGSA or each particle in PSO), the classification accuracy (CA) is evaluated with 10-fold cross-validation. In addition, the number of channels (no_ch) is evaluated in this step. NGSA or PSO then use these values to evolve the population. To compare the effectiveness of various classification techniques, we considered using RF, DA, SVM, or KNN classifiers.
6. The processes in the third to fifth steps are repeated, generating a progressively evolving population until the termination criterion has been reached.

The process is terminated after reaching a predefined number of iterations, MaxIter=50. The population size was set to 100. All operations presented in Figure 5 were implemented by Matlab 2022. 

## 3. Results and Discussion

To emphasize the impact of EEG channel reduction, we start by presenting the classification results using all 19 channels (full-channel), followed by results after applying channel selection techniques.

### 3.1. Full-Channel-Based Results

In this part, the results of classifying MCI against HC using all EEG channels are presented. In this case, no optimization was applied to optimize channels, and the classifiers’ parameters were manually selected. As previously mentioned, each channel’s signal was filtered with a band-pass filter ranging from 0.5 to 32 Hz. The filtered signal was then divided into 3660 non-overlapping segments (M = 3660), with each segment being 10 s long. Out of these segments, 1740 were from patients with MCI, while 1860 segments were from healthy control subjects. Each segment of the 19 EEG channels was transformed into a feature vector of length 114 using one of the adopted feature extraction (FE) methods. The resulting feature matrix had dimensions of 3660 × 114 and was then inputted into the classifier. Table 1 displays the classification outcome for each of the seven FE methods using the KNN classifier with k = 3. The 10-fold CV strategy calculated each result value in the table as the average of ten performance values. The findings show that the two methods with the highest performance, DW+ThEn and DWT+LogEn, had average classification accuracies of 99.89% and 99.84%, respectively. Additionally, the DWT+ShEn and DWT+SuEn FE methods showed classification accuracies greater than 99%. In contrast, the methods that performed the worst were DWT+Eng and DWT+NoEn.

**Figure 6:**
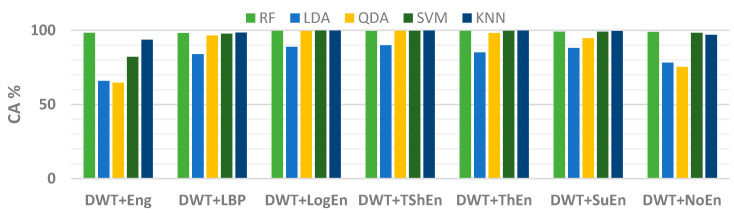
The classification accuracy with five classifiers (full-channel-based results).

Furthermore, four other classification algorithms were examined alongside KNN. The accuracy scores of the RF (with a tree depth of 30), LDA, QDA, and SVM (with a polynomial kernel) can be seen in Figure 6. The figure illustrates that the classification accuracy scores produced by DA classifiers were the lowest. Additionally, the figure demonstrated that out of all the classifiers, the DWT+Eng and DWT+NoEn FE methods did not perform well compared to the others. Thus, these methods were not investigated further. Next, we studied the classifiers’ performance after implementing channel selection and parameter optimization.

### 3.2. Selected-Channel-Based Results

Here, the classification results based on the channel subsets selected using the proposed channel selection methods are presented. To illustrate the need for effective EEG selection methods, we first present results when the channels were selected using a simple method, incremental evaluation. In this case, single-channel-based classification was evaluated. This produced a score for each channel that signifies its importance when it was used individually. Subsets of two or more channels can then be formed by selecting the channels based on their individual scores. Figure S1a (in the Supplementary Materials) displays the single-channel-based classification accuracy scores with the DWT+LBP and DWT+LogEn FE methods. Figure S2b displays the achieved accuracy scores based on the incremental evaluation. The figure demonstrates that this simple channel selection method could not exceed the accuracy obtained using all 19 channels (see Table 1). In the following sections, we present the outcomes of the proposed channel selection techniques. 

#### 3.2.1. Greedy-Algorithm-Based Channel Selection Results

**Figure 7:**
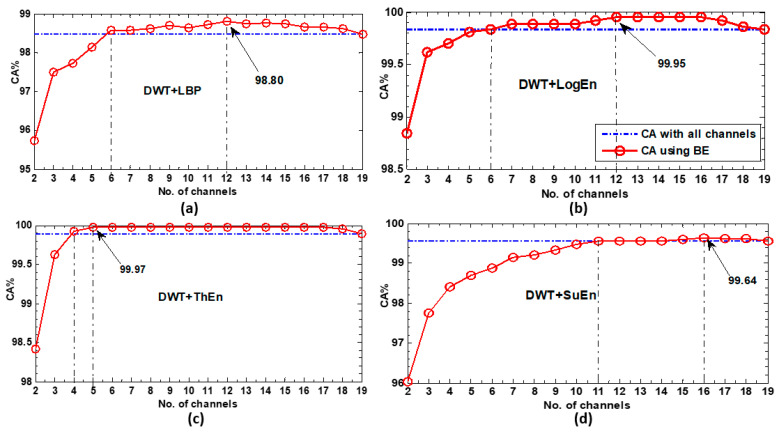
The KNN classification results of the BE-based selected channels for each FE method. (**a**) DWT+LBP, (**b**) DWT+LogEn, (**c**) DWT+ThEn, and (**d**) DWT+SuEn.

Figure 7 illustrates the classification accuracy scores obtained when using the back-elimination (BE) algorithm to select EEG channels. The KNN classifier was employed for classification, while four DWT-based methods were utilized for feature extraction. In the figure, the blue line represents the accuracy achieved when the 19 channels were included in the classification. The red curve indicates classification scores based on the BE algorithms. At each iteration of the BE, there was an accuracy value (local optimum) represented by a red circle. Surprisingly, the outcomes revealed that using a lower number of selected channels led to higher accuracy scores compared to using all 19 channels. For example, in Figure 7a, the accuracy score achieved with all 19 channels was 98.47%. However, by applying BE with the DWT+LBP FE method, this score was surpassed by just six selected channels. With this FE method, a maximum score of 98.80% was attained with 12 channels. Even better outcomes were observed with the DWT+LogEn, as shown in Figure 7b. Furthermore, the most remarkable results were obtained with the DWT+ThEn FE method, as depicted in Figure 7c. Using only five selected channels, an accuracy of 99.97% was attained, surpassing the accuracy achieved with all channels. In addition, just four channels were needed to exceed the accuracy achieved with all EEG channels. 

The classification accuracy results obtained when using the FA algorithm to select EEG channels are presented in Figure S2. Similar to the BE algorithm, FA required fewer channels to achieve the same accuracy as that obtained with all channels across all FE methods. Specifically, the DWT+LBP, DWT+LogEn, DWT+ThEn, and DWT+SuEn FE methods surpassed the full-channel-based score when 8, 6, 7, and 10 channels were selected, respectively. In summary, the results demonstrate that the BE and FA algorithms can effectively select a smaller subset of EEG channels without compromising classification accuracy, and in some cases, even outperformed the accuracy achieved when using all channels. 

#### 3.2.2. Results Using Multi-Objective Optimization Methods

With the adopted multi-objective optimization methods, the most relevant channels were selected. Simultaneously, these methods optimized the classifier parameters. The objective was to optimize the selection of channels by minimizing their number while maximizing accuracy.

##### NSGA-Based Results

**Figure 8:**
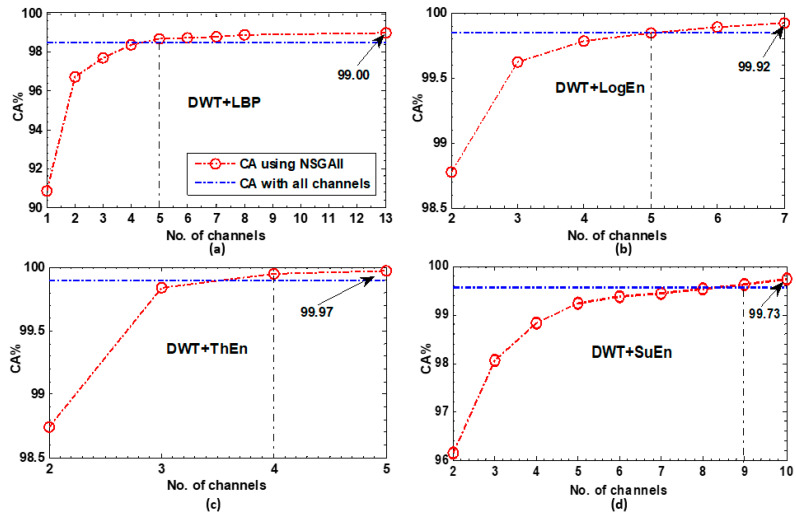
The KNN classification results of the NSGA-II-based selected channels for each FE method. (**a**) DWT+LBP, (**b**) DWT+LogEn, (**c**) DWT+ThEn, and (**d**) DWT+SuEn.

The classification accuracy scores of the KNN classifier using four different FE methods and the NSGA-II algorithm for EEG channel selection are depicted in Figure 8. As indicated in the figure, NSGA-II successfully identified a smaller number of channels that resulted in achieving and even surpassing the accuracy score achieved using all available channels. For the DWT+LBP and DWT+LogEn FE methods, five channels were sufficient to achieve the full-channel-based score. The DWT+ThEn FE method achieved this score with just four channels, while the DWT+SuEn FE method required ten channels. As shown in Figure 8, the maximum classification accuracies were achieved with channel subset sizes of 13, 7, 5, and 10 for the four FE methods. Notably, the DWT+ThEn and DWT+LogEn were the most effective in attaining the highest scores with the channels’ least number, as observed in Figure 8b,c. Furthermore, the results indicate that the choice of feature extraction methods significantly influenced the performance of the NSGA-II algorithm. For instance, the DWT+ThEn and DWT+LogEn not only obtained the highest accuracy scores with NSGA-II but also attained the highest full-channel-based scores, 99.89% and 99.84%, respectively (refer to Table 1).

Figure S3 presents the classification results based on the channel subsets selected using NSGA-III. The results demonstrate that NSGA-III achieved a comparable accuracy level to all channel methods using a smaller number of channels. Similar to NSGA-II, NSGA-III successfully attained and surpassed the full-channel-based accuracy. For DWT+LBP, DWT+LogEn, and DWT+ThEn, NSGA-III achieved full-channel accuracy with six, five, and four channels, respectively. The maximum classification accuracy values were obtained with these three FE methods, corresponding to channel numbers 9, 11, and 5, respectively. 

##### PSO-Based Results

**Figure 9:**
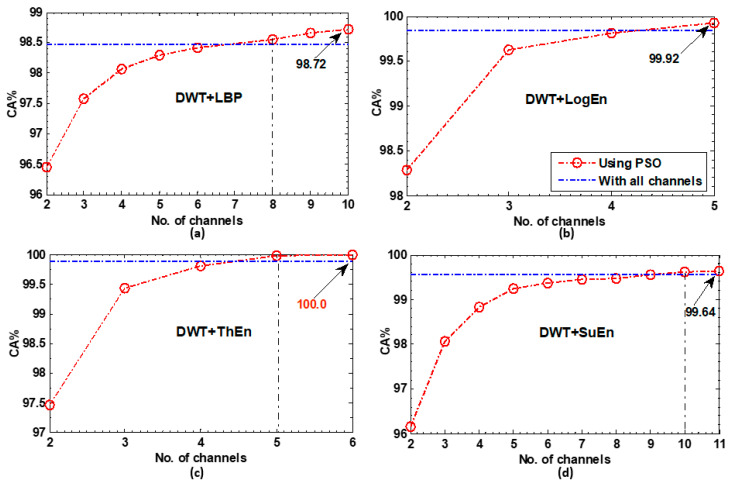
The KNN classification results of the PSO-based selected channels for each FE method. (**a**) DWT+LBP, (**b**) DWT+LogEn, (**c**) DWT+ThEn, and (**d**) DWT+SuEn.

Figure 9 presents the results when channel selection optimization was performed by the multi-objective PSO. Over all the FE methods, the results show that, with a smaller number of channels, PSO successfully attained and surpassed full-channel-based accuracy. For DWT+LBP, eight channels were required to attain full-channel-based accuracy, whereas 10 channels yielded maximum accuracy, as shown in Figure 9a. A better result is displayed in Figure 9b, where the features extracted using DWT+LogEn exceeded the full-channel accuracy with just five channels. With DWT+ThEn (see Figure 9c), full-channel accuracy was exceeded with only five channels, and perfect 100% accuracy was achieved using six channels. Similarly, to other channel selection techniques (BE, FA, NSGA-II, and NSGA-III), the DWT+ThEn feature extraction method achieved the best results with PSO.

In summary, the results obtained in Figure 7, Figure 8 and Figure 9 indicate the possibility of selecting a small number of channels that yielded superior classification accuracy compared to employing all available channels. This was due to the presence of insignificant channels that could be redundant and have a negative impact on the classification’s accuracy.

#### 3.2.3. Evaluation of EEG Channel Selection Approaches

**Figure 10:**
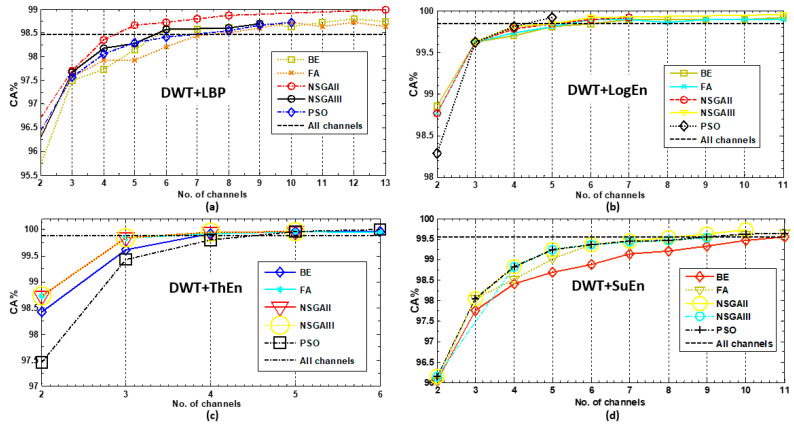
Performance comparisons of the investigated channel selection approaches for each FE method. (**a**) DWT+LBP, (**b**) DWT+LogEn, (**c**) DWT+ThEn, and (**d**) DWT+SuEn.

Here, the results obtained using the greedy algorithms and the multi-objective optimization algorithms are analyzed and discussed. Figure 10 provides the KNN-based classification accuracy scores achieved by these algorithms, considering various feature extraction methods. For instance, with the results for the DWT+LBP FE method (Figure 10a), the results indicate that the multi-objective optimization methods generally outperform the greedy methods across most channel subset sizes. The accuracy achieved when using all channels was 98.47%. However, all selection methods, including BE, FA, NSGA-II, NSGA-III, and PSO, achieved and surpassed this accuracy with 6, 8, 5, 6, and 8 channels, respectively.

As a summary of Figure 10, the results reveal that multi-objective optimization methods consistently outperformed greedy algorithms across different feature extraction methods. NSGA-II, NSGA-III, and PSO show excellent performance, achieving high accuracies with a smaller subset of channels. The specific performance varied depending on the feature extraction method utilized. Figure S4 shows the channel topographies for the best solution (the optimal channel subset) for each feature extraction method, which resulted in the highest classification accuracy.

**Figure 11:**
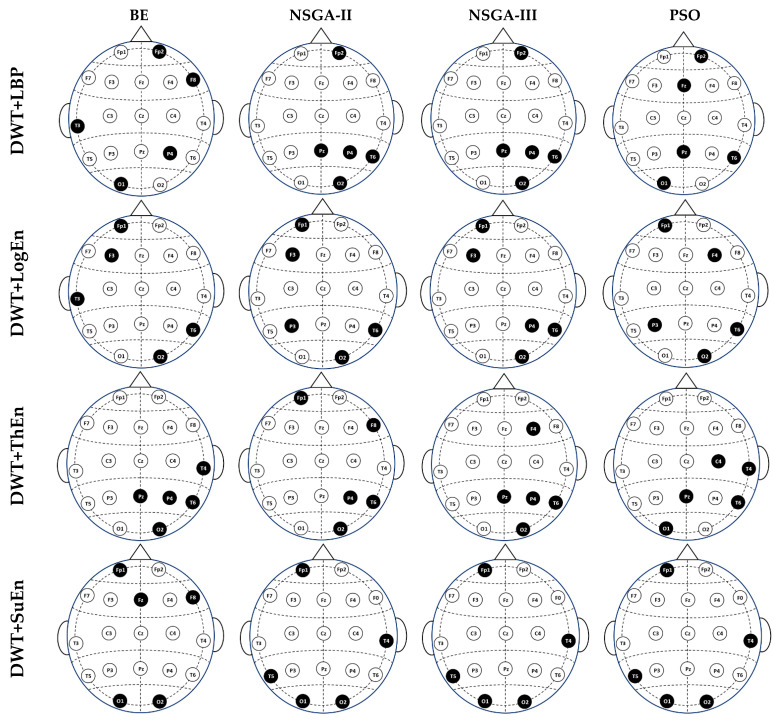
The returned five-channel solutions.

For a more channel-topographies-based discussion, Figure 11 displays the five-channel solutions (subsets of five channels) obtained by the BE, NSGA-II, NSGA-III, and PSO selection methods for each feature extraction method. When examining each row (independently for each feature extraction method), it can be observed that the selected channels in the four solutions did not match exactly. This is usually because each selection method has a different way of generating solutions. However, there were still some overlapping channels. For example, when considering the DWT+LogEn-based solutions, Fp1, T6, and O2 existed in the subsets created by BE, NSGA-II, NSGA-III, and PSO. Similarly, when examining each column in Figure 11 (independently for each selection method), it is observed that the subset varied with the used feature extraction method. The reason behind this is that for a given feature extraction method, unique biomarkers were extracted, resulting in a varying set of selected channels. The results reported in [27] also confirm that the choice of channels is contingent upon the feature extraction approach employed. In [27], the authors employed Fisher’s approach to choose the features obtained from four-relative power methods. The authors used these methods, all based on relative power, for feature extraction. The results in [27] demonstrated the selection of a different optimum channel subset for each FE method. For instance, their second feature extraction method selected five channels that differed from those selected by the third and fourth feature extraction methods. It is worth noting that certain channels appeared in most solutions across all feature extraction methods, such as T6 and O2. These channels consistently contributed to achieving optimal solutions, regardless of the specific feature extraction method or selection algorithm employed.

**Figure 12:**
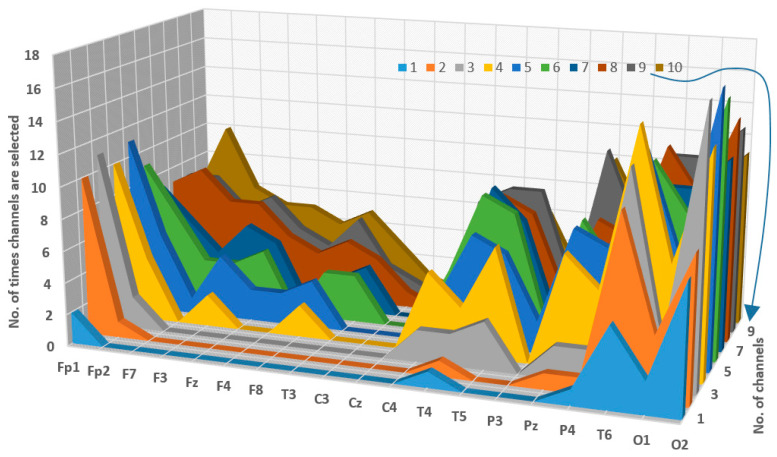
The frequency of selection for EEG channels across all channel selection approaches and feature extraction methods.

To get an idea of which channels are the most and least important, Figure 12 was created. The figure highlights the frequency with which each channel was selected in each channel subset (subset of two channels, subset of three channels, …, subset of 10 channels). From the figure, it is evident that O2, T6, and Fp1 were consistently selected as the most frequently chosen channels within the subset of 1–5 channels. Additionally, O1, Pz, T5, and T4 were also commonly selected across various subsets. Conversely, channels such as C3, P3, F4, and T3 were less frequently chosen compared to others. Furthermore, channel Cz was not selected by any subset, indicating its lack of contribution in the channel selection process.

#### 3.2.4. Performances of Classifiers

In this subsection, RF, DA, SVM, and KNN classification accuracies are compared when NSGA-II was applied for channel selection. Additionally, NSGA-II was employed to select the classifier parameters through the variable Param, as discussed in Section 2.5.2. The findings demonstrate that the selected subset of channels affected the selected value of Param. For instance, with the KNN, when only one channel was selected, the number of neighbors was set to 3 or 5 (k = 3 or 5). However, K was set to 1 as the subset size increased. When the subset size was small, the RBF kernel was used in SVM. On the other hand, when the subset size was large, the polynomial kernel was used. In the RF classifier, the selected tree depth values ranged from 29 to 35 across all cases. When it comes to DA, the quadratic type was consistently used, irrespective of the subset size.

**Figure 13:**
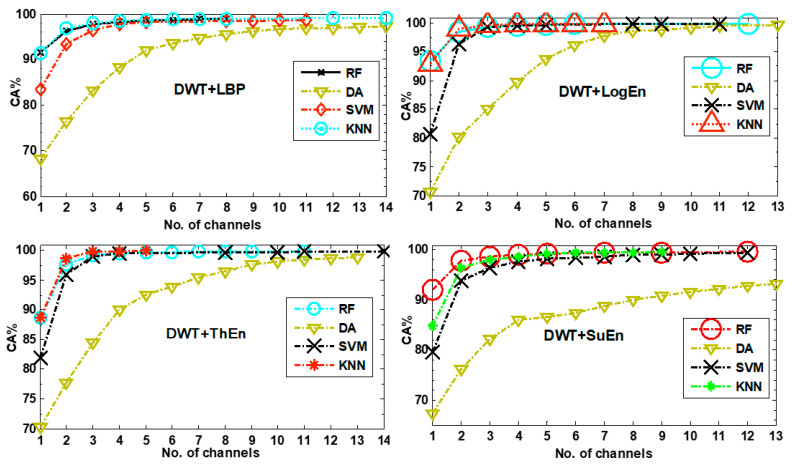
The NSGA-II-based classification results with four classifiers.

Figure 13 displays the classifiers’ classification results for each FE method. The figure shows that KNN and RF classifiers had similar performances and achieved the best results, while DA classifiers achieved the worst results with all FE methods. In general, the performance of SVM was better than that of DA but worse than that of KNN and RF. With all FE methods, the performance of SVM improved as the subset size increased. In this case, SVM achieved accuracy scores close to those of KNN and RF. The RF classifier achieved its highest accuracy of 99.97% with the DWT+ThEn FE method on 11 channels. However, surprisingly, the KNN classifier attained a perfect accuracy, 100%, with the same FE method, but on a subset of five channels. DWT+LogEn, QDA, and SVM achieved their highest accuracies (99.62% and 99.95%) using 13 and 11 channels, respectively. The results also show that the classifiers improved when their parameters were selected by NSGA-II. For example, DWT+LogEn+KNN achieved 99.95% accuracy with 11 channels using 3-NN (k = 3). When NSGA-II chose k = 1, it achieved the same accuracy with seven channels. Another example is DWT+ThEn+KNN, which achieved 100% accuracy by utilizing six channels of 3-NN and five channels of 1-NN.

### 3.3. Discussion with the Literature

The present study introduced several DWT-based methods for MCI detection in two scenarios: full-channel-based classification and selected-channel-based classification. In the first scenario, DWT-based features extracted from all available channels were included in the classification. Conversely, in the second scenario, the classification only included features from selected channels. We employed three multi-objective optimizations and two greedy algorithms for channel selection. In both scenarios, different classifiers were employed to classify MCI and HC features. The two scenarios were considered to be discussed with the studies in the literature, considering the used dataset.

In terms of full-channel classification, most studies in the literature [23,26,29,30,32,33,34,35,36,40] used the available channels in the data at hand. Some of these studies [29,30,32,34,40] achieved high classification accuracy scores of 98.78%, 96.41%, 99.40%, 97.60, and 99.51, respectively. The present study outperformed these scores. Table 1 shows that the features extracted by DWT+LogEn, DWT+TShEn, and DWT+ThEn achieved classification scores of 99.84%, 99.81%, and 99.89%, respectively. This outperformance indicates that the proposed DWT-based features are more reliable for MCI detection.

In terms of selected-channel classification, there are attempts in [24,25,27,28,31] to reduce the quantity of channels. The studies [24,25] divided the scalp area into five subareas and conducted classification using these subareas. The study [24] achieved comparable accuracy of 88.89% for all subareas, with the exception of the frontal subarea, which displayed a lower accuracy score. The study [25] used the same dataset as the reference [24]. The left-temporal subarea yielded the highest accuracy of 88.9%. The objective of the present study was to determine the most relevant channels in different locations of the scalp, in line with prior studies [27,28,31]. Fisher’s class separability criterion was used by the authors in [27] to find the best electrodes (channels) and frequency subbands for getting the most responsive relative power features. From a total of thirty channels, five were chosen from distinct brain regions (Fp2, F7, T3, T5, and T6), resulting in a maximum classification accuracy of 90.25% when employing the SVM classifier. The study [28] investigated channel reduction by utilizing the incremental evaluation methodology to determine the best subset of channels. Only when all 19 channels were included for classification was the maximum accuracy of 96.94% was achieved. The present study also verified that the incremental evaluation method (refer to Figure S1) is ineffective in choosing a lower number of channels that can achieve higher accuracy compared to employing all channels. In [31], the researchers manually selected channel subsets, limited to symmetric combinations of two, four, six, and eight channels, to assess the classification accuracy. An accuracy of 86.85% was achieved by using a symmetrical combination of eight channels, which was the maximum accuracy obtained. By focusing solely on symmetric channel pairs, the analysis disregards alternative channel combinations that could potentially yield higher accuracy scores.

The current study utilized heuristic optimization approaches to address these restrictions. We used several types of multi-objective optimization (NSGA-II, NAGA-III, and MOPSO) in this study to find the best combinations of EEG channels and classifier parameters. Furthermore, we compared the results of these methods with those obtained via greedy algorithms. The results from Figure 8, Figure 9, and Figure S3 indicate that the multi-objective optimization approaches were able to find a few appropriate channels. This made them more accurate than classifiers that used all channels. Based on the findings presented in Figure 11 and Figure 12, it can be concluded that the most effective channels are located in distinct brain regions. This supports the findings of a previous study [24,27], which indicated that many brain regions are impacted in patients with MCI.

To evaluate the significance of this study, it is valuable to compare the findings of the methodologies investigated in this study with those in the literature. A summary of the current findings in comparison to the results reported in previous studies that used the same sourced dataset is presented in Table 2. At first, we looked at the DWT-based FE methods that were used with all 19 channels (full channel-based classification). The classification accuracy scores that were obtained were higher than those found in previous studies. Furthermore, we enhanced the results by selecting the most relevant channels using multi-objective optimization algorithms (selected channel-based classification). The NSGA-II algorithm selects only five channels, achieving perfect accuracy with the DWT+ThEn+KNN combination.

## 4. Conclusions and Future Work

This study introduces efficient DWT-based methods to detect MCI from EEG signals. These methods involve decomposing each channel’s signal into a set of distinct frequency band signals, then extracting features using a non-linear measure. To ensure a comprehensive investigation, various measures and classifiers were employed, resulting in several feature extraction and classification combinations. These combinations were evaluated in two scenarios: a full-channel-based experiment and a selected-channel-based experiment. In the first experiment, DWT-based features were extracted from all available EEG channels and then sent for classification. On the other hand, in the second experiment, only features belonging to the selected channels were included for classification. For channel selection, this study employed three multi-objective optimizations (NSGA-II, NSGA-III, and MOPSO) and two greedy algorithms (FA and BE).

The full-channel-based results show that the proposed DWT-based features were reliable for MCI detection, and the obtained classification scores outperformed those reported in the literature. Furthermore, the classification accuracy scores were further improved when fewer suitable channels were selected. For instance, the combination of DWT, ThEn, and KNN yielded a full-channel accuracy of 99.84% in classifying MCI. This combination attained an accuracy of 99.92% using only four channels that were selected by FA and BE. NSGA-II, NSGA-III, and PSO all obtained an accuracy of 99.97% with the same quantity of channels. Interestingly, NSGA-II achieved a perfect accuracy of 100% with only five selected channels, whereas PSO attained the same accuracy by selecting six channels. 

Overall, the results indicate that using efficient techniques, particularly those based on multi-objective optimization, to choose the most valuable EEG channels leads to increased accuracy. This suggests that certain channels may contain duplicate, irrelevant, or inferior information. The results also indicate that the most effective channels are located in several regions of the brain (frontal, parietal, temporal, and occipital). In addition, the channels that are selected are influenced by other aspects, such as the techniques used for extracting features, the classifiers and their parameters, and the methodology employed for channel selection. Although the results are promising, they should be verified by more methods for feature extraction and EEG channel selection with a larger dataset. The authors also intend to verify and validate the suggested approaches in this study with other neurological conditions.

## Figures and Tables

**Table 1:** The KNN classification results (mean ± standard deviation) using all channels (full-channel).

FE Methods	Accuracy (%)	Sensitivity (%)	Specificity (%)	F-Score (%)
DWT+Eng	93.77 ± 1.60	93.57 ± 2.11	93.97 ± 1.40	93.45 ± 1.66
DWT+LBP	98.47 ± 0.74	98.90 ± 0.58	98.10 ± 1.08	98.38 ± 0.79
DWT+LogEn	99.84 ± 0.26	99.94 ± 0.18	99.74 ± 0.50	99.83 ± 0.28
DWT+TShEn	99.81 ± 0.18	100.0 ± 0.00	99.64 ± 0.35	99.80 ± 0.19
DWT+ThEn	99.89 ± 0.19	100.0 ± 0.00	99.79 ± 0.36	99.88 ± 0.20
DWT+SuEn	99.56 ± 0.37	99.60 ± 0.39	99.53 ± 0.45	99.54 ± 0.39
DWT+NoEn	97.08 ± 0.73	97.08 ± 1.38	97.10 ± 0.74	96.92 ± 0.76

**Table 2:** A summary of the outcomes of earlier studies that employed the same sourced dataset and this study regarding the MCI vs. HC classification.

Study	Feature Extraction Methods	Classifiers	No. of Channels	CA (%)	Validation Type	
[24] 2016	Power, ratio power, and relative power for different bands	Neurofuzzy +KNN	3	88.89%	Hold-out	
[25] 2019	Correlation-based label consistent K-SVD with spectral features		3	80%	Hold-out	
[26] 2019	Power spectral-based features	KNN	19	81.5 %	Not determined	
[28] 2019	SWT + statistical features	SVM	19	96.94%	Based on intra-subject validation	
[29] 2020	Auto-regressive and permutation entropy	ELM	19	98.78%	10-fold CV	
[30] 2022	--	LSTM	19	96.41%	Five-fold CV	
[32] 2022	Spectral, functional connectivity, and nonlinear features	SVM	19	99.4%	10-fold CV	
[33] 2023	DWT leader	AdaBoostM1	19	93.50%	10-fold CV	
[34] 2023	EMD + Log energy entropy	KNN	19	97.60%	10-fold CV	
[40] 2024	VMD+LogEn	KNN	19	99.51%	10-fold CV	
			11	99.64%	10-fold + NSGA-II	
Present study	DWT+LogEn	KNN	19	99.84%		10-fold CV
	DWT+ThEn			99.89%		
	DWT+LogEn	KNN	7	99.95%		10-fold + NSGA-II (PSO)
	DWT+ThEn		5 (6)	100%		

## Data Availability

Publicly available datasets are used in this study and can be downloaded from: https://misp.mui.ac.ir/en/eeg-data-0, accessed on 1 April 2023.

## Supplementary Materials

The following additional results can be downloaded at https://www.mdpi.com/article/10.3390/diagnostics14151619/s1, Figure S1: The KNN classification accuracy based on (a) single-channel and (b) incremental evaluation; Figure S2: The KNN classification results of the FA-based selected channels for each feature extraction method. (a) DWT+LBP, (b) DWT+LogEn, (c) DWT+ThEn, and (d) DWT+SuEn; Figure S3: The KNN classification results of the NSGAIII-based selected channels for each FE method. (a) DWT+LBP, (b) DWT+LogEn, (c) DWT+ThEn, and (d) DWT+SuEn; Figure S4: The channel topographies for the best solution for each feature extraction method. (a) DWT+LBP, (b) DWT+LogEn, (c) DWT+ThEn, and (d) DWT+SuEn.
