# Supplementary material for: EEG-Based Detection of Mild Cognitive Impairment Using DWT-Based Features and Optimization Methods
Source: Diagnostics (Basel). 2024 Jul 26;14(15):1619. doi: 10.3390/diagnostics14151619 (PMC11312237; doi:10.3390/diagnostics14151619)
Supplement: Supplementary file 1 [file diagnostics-14-01619-s001.zip › diagnostics-3078259-supplementary.pdf]

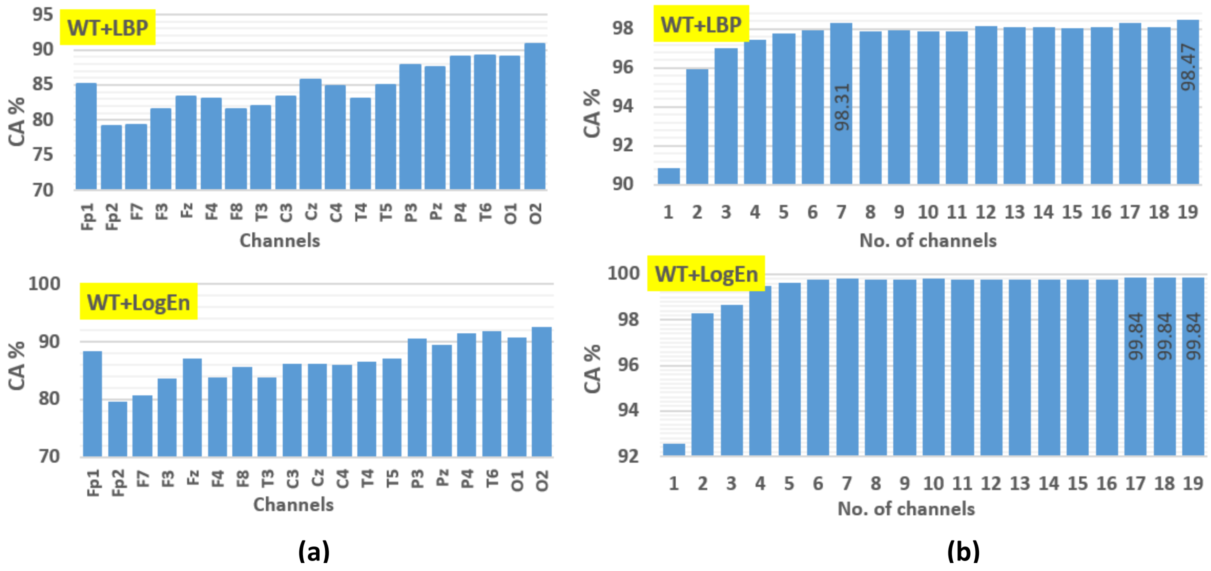

Figure S1: The KNN classification accuracy based on (a) single-channel and (b) incremental evaluation.

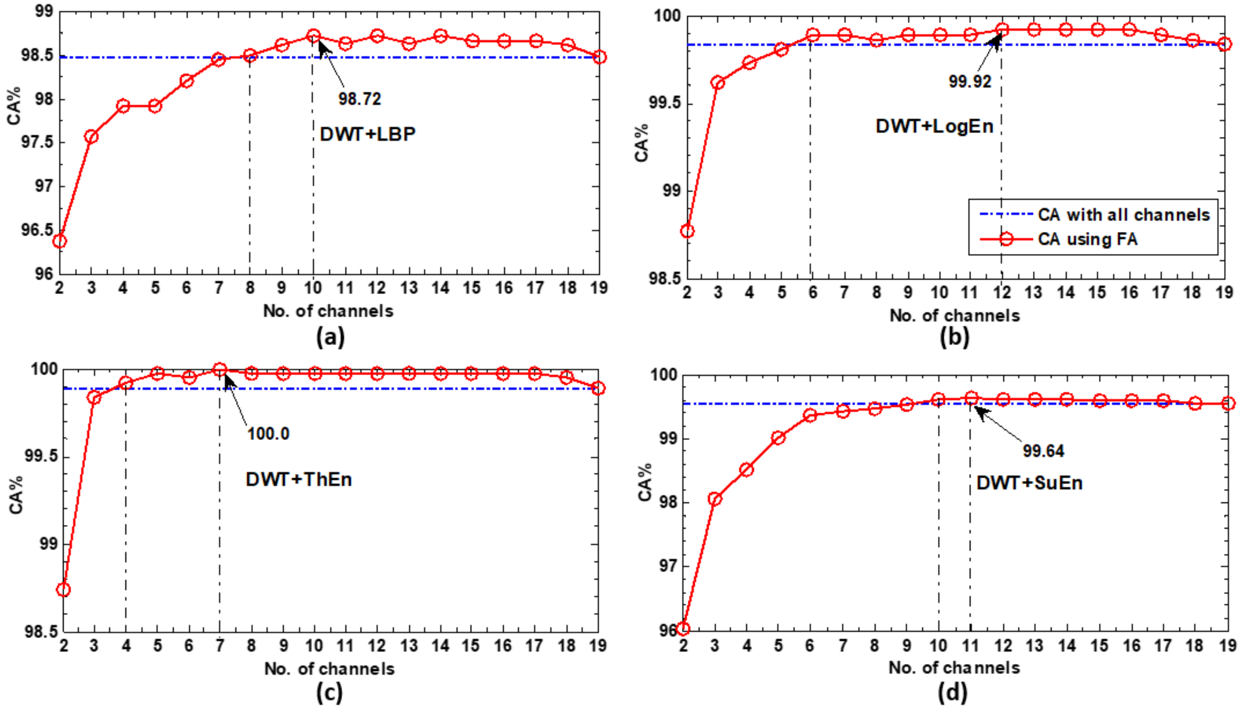

Figure S2: The KNN classification results of the FA-based selected channels for each feature extraction method. (a) DWT+LBP, (b) DWT+LogEn, (c) DWT+ThEn, and (d) DWT+SuEn.

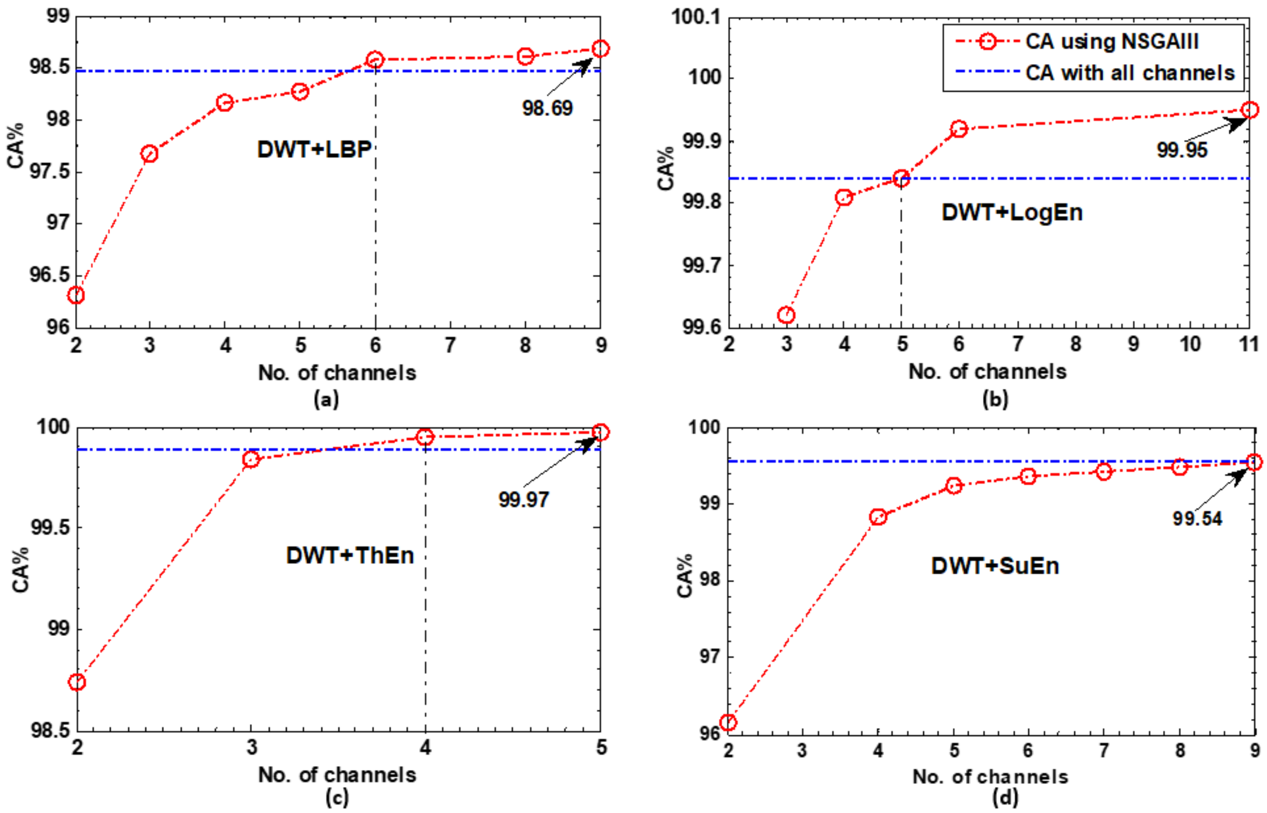

Figure S3: The KNN classification results of the NSGAIII-based selected channels for each FE method. (a) DWT+LBP, (b) DWT+LogEn, (c) DWT+ThEn, and (d) DWT+SuEn.

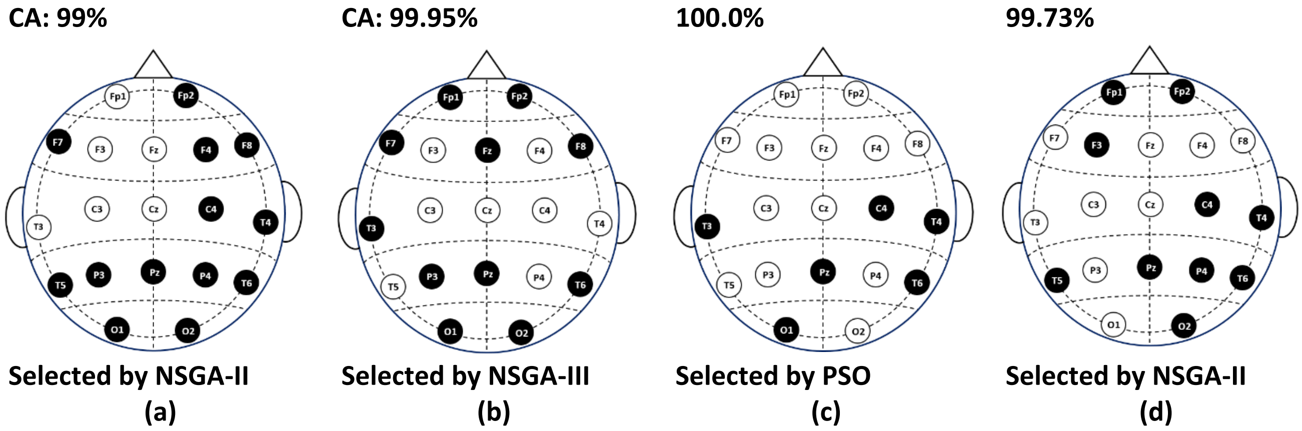

Figure S4: The channel topographies for the best solution for each feature extraction method. (a) DWT+LBP, (b) DWT+LogEn, (c) DWT+ThEn, and (d) DWT+SuEn.
